# Pyroptosis and polarization of macrophages in septic acute lung injury induced by lipopolysaccharide in mice

**DOI:** 10.1002/iid3.1197

**Published:** 2024-03-19

**Authors:** Sijiang Zhou, Xia Yang, Kanglin Mo, Zong Ning

**Affiliations:** ^1^ Department of Emergency the First Affiliated Hospital of Guangxi Medical University Nanning Guangxi China; ^2^ Department of General Medicine the First Affiliated Hospital of Guangxi Medical University Nanning Guangxi China; ^3^ Department of Respiratory Medicine the First Affiliated Hospital of Guangxi Medical University Nanning Guangxi China

**Keywords:** acute lung injury, alveolar macrophage, polarization, pyroptosis, sepsis

## Abstract

**Background:**

Pyroptosis and polarization are significant contributors to the onset and development of many diseases. At present, the relationship between pyroptosis and polarization in acute lung injury (ALI) caused by sepsis remains unclear.

**Methods:**

The ALI model for sepsis was created in mice and categorized into the blank control, lipopolysaccharide (LPS) group, LPS + low‐dose Belnacasan group, LPS + high‐dose Belnacasan group, LPS + low‐dose Wedelolactone group, LPS + high‐dose Wedelolactone group, and positive control group. The wet‐dry specific gravity was evaluated to compare pulmonary edema. Hematoxylin–eosin, Masson, and terminal deoxynucleotidyl transferase dUTP nick end labeling staining techniques were conducted to observe and contrast the pathological changes in lung tissue. ELISA was utilized to identify M1 and M2 macrophages and correlated inflammatory factors. Immunohistochemical staining and flow cytometry were employed to identify markers of M1 and M2 macrophages in lung tissue. Propidium iodide staining, together with flow cytometry, was utilized to observe the degree and positive rate of pyroptosis of alveolar macrophages. Western blot analysis was conducted to detect the expression levels of Caspase 1, Caspase 11, GSDMD, and IL‐18 in the lung tissues of each group. The real‐time quantitative polymerase chain reaction method was used to ascertain relative expression levels of NLRP3, Caspase 1, Caspase 11, GSDMD, IL‐18, iNOS, and Arg‐1 in lung tissues of all groups.

**Results:**

In mice with sepsis‐induced ALI, both classical and nonclassical pathways of pyroptosis are observed. Inhibiting pyroptosis has been found to ameliorate lung injury, pulmonary edema, and inflammation induced by LPS. Notably, the expression of NLRP3, Caspase 1, Caspase 11, GSDMD, IL‐1β, IL‐18, TGF‐β, CD86, CD206, iNOS, and Arg‐1 were all altered in this process. Additionally, alveolar macrophages were polarized along with pyroptosis in mice with ALI caused by sepsis.

**Conclusion:**

Pyroptosis of alveolar macrophages in the context of ALI in mice infected with sepsis has been linked to the polarization of alveolar macrophages toward type M1.

## 1 INTRODUCTION

Sepsis is a life‐threatening multiorgan dysfunction caused by the host's innate immune response to bacterial, fungal, or viral infection.^1^ Acute lung injury (ALI) may arise during the early stages of sepsis. The etiology of ALI is intricate, and up‐to‐date findings have indicated that inflammation is a crucial mechanism in its onset. The pivotal role of lipopolysaccharide (LPS), a major constituent of endotoxin, in the emergence of ALI is well established.^3^

Pyroptosis is a newly discovered type of programmed cell death that is initiated by inflammatory stimuli and executed by the Gasdermin protein. Presently, it is believed that pyroptosis primarily takes place via the classical pyroptosis pathway that is reliant on Caspase 1, as well as the nonclassical pyroptosis pathway mediated by Caspase 4/5/11.^4^

As a significant constituent of innate immunity, macrophages bear a critical responsibility in upholding the immune surroundings of the body.^5^ Macrophages exhibit remarkable plasticity, allowing for changes in physiological function according to variations in the microenvironment in vivo and in vitro, resulting in the production of distinct cell populations. This process is referred to as polarization.^6^ Natural macrophages, referred to as M0 type, exhibit diverse functions upon polarization into macrophages with varying phenotypes.^7^ Based on differences in phenotype and secreted cytokines, macrophages may be categorized as either classically activated macrophages (M1 type, also referred to as proinflammatory phenotype) or replacement‐activated macrophages (M2 type, also known as anti‐inflammatory phenotype).^7^ In our prior in vitro investigations, we have confirmed that M0, M1, and M2 macrophages exhibit pyroptosis, with M1 macrophages having the most substantial effect, followed by M2 macrophages. These findings are significant in establishing the contribution of diverse macrophage phenotypes in the inflammatory response.^8^ Great strides have been taken in understanding the pathogenesis of pyroptosis, leading to the discovery and utilization of relevant inhibitors. Nonetheless, the extraordinary plasticity of macrophages makes them highly responsive to even subtle changes in both intra‐ and extracellular environments. This responsiveness can lead to macrophage polarization, imbalances in phenotype, distribution, and proportion, as well as alterations in the secretion of inflammatory factors, all of which can culminate in damage to the body. However, the regulatory mechanism of this phenomenon has yet to be clarified. Therefore, drawing on previous research, we have established a mouse model of LPS‐induced ALI in sepsis, and aim to investigate in vivo the interaction mechanism between polarization and pyroptosis. Our aim is to regulate the occurrence and progression of pyroptosis to influence the direction of polarization and decrease the resulting harm to the body. Through this research, we intend to provide a theoretical foundation for the immunoregulatory treatment of ALI with sepsis.

## 2 MATERIALS AND METHODS

### 2.1 Animals

Based on pre‐experimentation, 56 C57BL/6 mice, 20 ± 2 g, 6–8 weeks, were provided with standard food and water while under observation at Guangxi Medical University, China. The Institutional Animal Care and Use Committee of Guangxi Medical University provided approvals for this study (approve number: No. 202106157).

### 2.2 Materials

Belnacasan (Cat. No. HY‐132O5)  purchased from MedChemexpress Biotechnology, USA; Wedelolactone (Cat. No. HY‐N0551) purchased from MedChemexpress Biotechnology, USA; Lipopolysaccharides (Product No. L2880) purchased from Sigma‐Aldrich. F4/80 Monoclonal Antibody purchased from BDPharmingen Biotechnology; PEAnti‐MouseCD86 purchased from BDPharmingen Biotechnology, USA; APCAnti‐mouseCD206 purchased from Invitrogen Biotechnology, USA.

### 2.3 Animal treatment and grouping

The mice were randomly divided into different groups: a vehicle (Control) group, an LPS group, an LPS + 6 mg/kg Belnacasan group (LPS + low‐Belnacasan), an LPS + 10 mg/kg Belnacasan group (LPS + high‐Belnacasan), an LPS + 6 mg/kg Wedelolactone group (LPS + low‐Wedelolactone), an LPS + 10 mg/kg Wedelolactone group (LPS + high‐Wedelolactone), an LPS + 10 mg/kg dexamethasone group (LPS + dexamethasone). Each group had *n* = 8. To establish ALI models, the mice were sensitized with LPS (10 mg/kg). The intervention group received a one‐time intraperitoneal injection of 6 mg/kg of Belnacasan and Wedelolactone solution, respectively, after an intraperitoneal injection of LPS for 1 h. The normal control group was administered a single intraperitoneal injection of an equal dose of normal saline, and the positive control group was given a one‐time intraperitoneal injection of 10 mg/kg of dexamethasone solution. Mice were euthanized via cervical dislocation following an 8‐h administration of normal saline, Belnacasan, Wedelolactone, and dexamethasone. If the mice die before 8 h, they need to be remolded for sampling.

### 2.4 Bronchoalveolar lavage fluid (BALF) and serum collection

Mice were anesthetized with 0.75% sodium pentobarbital. Thereafter, the heads of the mice were secured, the eyeballs were carefully removed with a set of forceps, and venous blood samples were collected. Subsequently, BALF was obtained by lavaging with phosphate‐buffered saline (PBS).

### 2.5 Enzyme‐linked immunosorbent assay

IL‐1β, IL‐18, and transforming growth factor‐β (TGF‐β) concentrations were assessed in the both BALF and serum obtained from all groups of mice via the use of ELISA kits (Shanghai Jianglai Biologicals, China). The manufacturer's guidelines were strictly followed for this analysis.

### 2.6 Determination of lung wet/dry ratio

After sacrificing the mice, the right upper lobe was rinsed thrice with physiological saline. Subsequently, the filter paper was patted down and weighed. Following a 72‐h drying period at 60°C, the dried mass was measured, and the lung wet/dry weight ratio was computed.

### 2.7 Lung histopathology

Mice were euthanized at 8 h postintervention for lung evaluation. One side of lung tissue was taken from each group, then washed three times with 0.9% saline and fixed in 4% paraformaldehyde. After 24 h of tissue fixation, it was dehydrated and embedded in paraffin. The tissue was later cut into 4 μm thick sections and treated with hematoxylin–eosin (HE) and terminal deoxynucleotidyl transferase dUTP nick end labeling (TUNEL) staining. Pathological changes in the tissue were then observed and evaluated under a microscope.

### 2.8 Immunohistochemical staining

The mice lung sections were subjected to immunohistochemical detection by incubating them overnight at 4°C with primary antibodies that were specific to F4/80 (diluted at 1:500), CD86 (diluted at 1:500), and CD206 (diluted at 1:500). The next day, all sections were washed with PBS and then incubated for 60 min with biotinylated goat anti‐mouse immunoglobulin at 4°C. Afterward, they were incubated for 10 min with streptavidin horse‐radish peroxidase at 37°C. The sections were then counterstained with hematoxylin and mounted. By following standard histochemical protocols for immunohistochemical staining, the stained sections were examined using an inverted microscope.

### 2.9 Propidium iodide (PI) staining and flow cytometry

The alveolar lavage fluid was used to extract alveolar macrophages, which were then counted. The volume was subsequently adjusted to 100 μL with PBS before adding 200 μL of a configured solution containing 0.05 mg/mL PI. The mixture was incubated for 15 min, protected from light, at room temperature. At the end of incubation, cell morphology and PI staining were observed under the inverted fluorescence microscope, and photographs were taken for comparison. Following this, the proportion of pyroptosis‐positive cells was determined using the flow cytometer. The utilized flow cytometers were fabricated by EXFLOW, belonging to the EXFLOW‐206/204/104 model series, with laser configurations comprising a 488 nm blue laser and a 638 nm red laser. The appropriate flow channel for PI staining was selected and FlowJo V10 software was used to analyze and record the resulting data.

### 2.10 Flow cytometry

Alveolar macrophages extracted from alveolar lavage fluid underwent cell counting and then resuspended in PBS for flow cytometry detection. Macrophage polarization was detected using F4/80‐FITC, CD86‐PE‐Cy7, and CD206‐APC antibodies. The cells were co‐incubated with primary antibodies for 30 min at room temperature and then washed twice with PBS. To detect staining from the flow antibody, select the corresponding channel and analyse the results using FlowJo V10 software. For forward scatter‐side scatter (FCS‐SSC) analysis, a two‐parameter scatter plot was generated to delineate major cell clusters, excluding cellular debris. Subsequently, PI‐SSC analysis produced a two‐parameter scatter plot within the lymphocyte gate, wherein positive cell clusters were identified as cells exhibiting compromised cell membranes in the samples. Furthermore, F4/80‐CD86 analysis involved the creation of a two‐parameter scatter plot within the lymphocyte gate, where Q2 represented a double‐positively expressing cell cluster corresponding to M1 cells. Similarly, a dual‐parameter scatter plot for F4/80‐CD206 within the lymphocyte portal was generated, with Q6 denoting a cluster of double‐positively expressing cells indicative of M2 cells.

### 2.11 Western blot

Proteins were extracted from lung tissue utilizing ice‐cold RIPA lysis buffer comprising protease inhibitors and phosphatase inhibitors. The samples underwent centrifugation at 12,000 rpm for 20 min at 4°C, and the supernatant was instantly collected. Separation of 30 μg of total protein per sample was carried out through sodium dodecyl sulfate‐polyacrylamide gel electrophoresis (SDS‐PAGE), followed by transferring them to PVDF membranes. The membranes were blocked with 5% skimmed milk for 2 h at room temperature. Afterward, they were incubated overnight at 4°C with anti‐Caspase 1 (1:500), anti‐Caspase 11 (1:500), anti‐GSDMD (1:500), anti‐IL‐18 (1:500), anti‐Vinculin (1:1000), and anti‐Cyclophilin B (1:1000) antibodies. Western blot analysis was performed according to the manufacturer's protocol, using an enhanced chemiluminescence detection reagent. To develop and analyze the density of immunoblotting bands, ImageJ was used.

### 2.12 Quantitative real‐time polymerase chain reaction (PCR)

RNA was extracted from lung tissues utilizing the Eastep Super Total RNA Extraction Kit. cDNA was synthesized using the Prime Script RT reagent Kit from Beijing Takara Biotechnology Co., Ltd. The Bio‐Rad iCyCler real‐time PCR7500 system with SYBR Green technology was used to quantify mRNA expression levels. Gene expression was analyzed quantitatively using the $2^{-\Delta \Delta C_{q}}$ method. The primer sequences were as follows: NLRP3 forward: 5′‐CCGCGTGTTGTCAGGATCTC‐3′, reverse:  5′‐AAGGGCATTGCTTCGTAGATAGA‐3′; Caspase‐1 forward: 5′‐CGTGGAGAGAAACAAGGAGTGGTG‐3′, reverse: 5′‐CTATCAGCAGTGGGCATCTGTAGC‐3′; Caspase‐11 forward: 5′‐GGTGGGAACTCTGGAGAAATGTGG‐3′, reverse: 5′‐ATCAATGGTGGGCATCTGGGAATG‐3′; GSDMD forward: 5′‐CTGGTGCTTGACTCTGGAGAACTG‐3′, reverse: 5′‐CTTGACAATAGGAACAGGGAGGCATAG‐3′; IL‐1β forward: 5′‐ AAATGCCACCTTTTGACAGTGA‐3′, reverse: 5′‐AAAGAAGGTGCTCATGTCCTCATCC‐3′; iNOS forward: 5′‐CCAAGCCCTCACCTACTTCC‐3′, reverse: 5′‐CTCTGAGGGCTGACACAAGG‐3′; Arg‐1 forward: 5′‐CTCCAAGCCAAAGTCCTTAGAG‐3′, reverse: 5′‐AGGAGCTGTCATTAGGGACATC‐3′; GAPDH forward: 5′‐TCACCATCTTCCAGGAGCGAGAC‐3′, reverse: 5′‐TGAGCCCTTCCACAATGCCAAAG‐3′. The conditions for PCR were 95°C for 30 s, followed by 40 cycles of 95°C for 15 s, and 60°C for 30 s.

### 2.13 Statistical analysis

Statistical analysis was conducted using IBM SPSS 26.0 software. The normality of the measurement data was evaluated using the Kolmogorov–Smirnov method, and the mean ± standard deviation was reported for normally distributed data. Independent Student's *t*‐test was applied to compare two groups, while one‐way analysis of variance was employed to compare multiple groups. The statistical significance was set at *p* < .05.

## 3 RESULTS

### 3.1 Pyroptosis inhibition alleviated lung pathological injury in LPS‐induced mice

**FIGURE 1:**
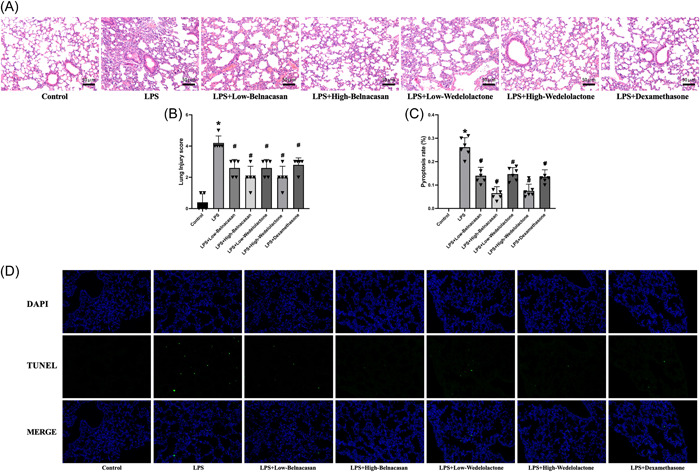
Pyroptosis inhibition alleviated lung pathological injury in  lipopolysaccharide (LPS)‐induced mice. (A) hematoxylin–eosin staining judged pathological alternations in lung tissues (×200), *N* = 3. (B) Comparison of lung tissue pathological score. (C, D) TUNEL assay detected the pyroptosis of lung cells in mice (×400), *N* = 6. *p < .05 versus control, ^#^ *p* < .05 versus LPS.

Initially, the comparison of lung tissue HE staining results demonstrates that the sepsis ALI group displays a noteworthy increase in inflammation and infiltration as compared to the blank control group. Conversely, the LPS + low Belnacasan, LPS + high Belnacasan, LPS + low Wedelolactone, LPS + high Wedelolactone, and LPS + dexamethasone groups, respectively, exhibit a notable decrease in the pathological status of lung tissues as compared to the LPS group (Figure 1A,B). In addition, TUNEL staining validates these outcomes (Figure 1C,D).

### 3.2 Pyroptosis inhibition reduced pulmonary edema in LPS‐induced mice

**FIGURE 2:**
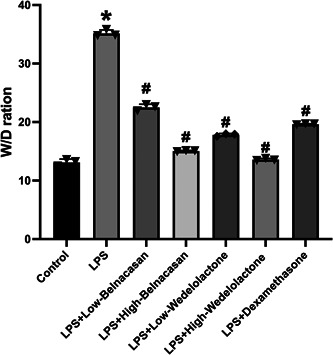
W/D ratio was measured to observe the change of pulmonary edema, *N* = 3. *p < .05 versus control, ^#^ *p* < .05 versus lipopolysaccharide.

Examination of the lung W/D ratio revealed an increase in lung edema in the LPS group in comparison to the control group. However, the LPS + low Belnacasan, LPS + high Belnacasan, LPS + low Wedelolactone, LPS + high Wedelolactone, and LPS + dexamethasone groups displayed a reduction in pulmonary edema relative to the LPS group (Figure 2).

### 3.3 Pyroptosis inhibition attenuated inflammatory response in LPS‐induced mice

**FIGURE 3:**
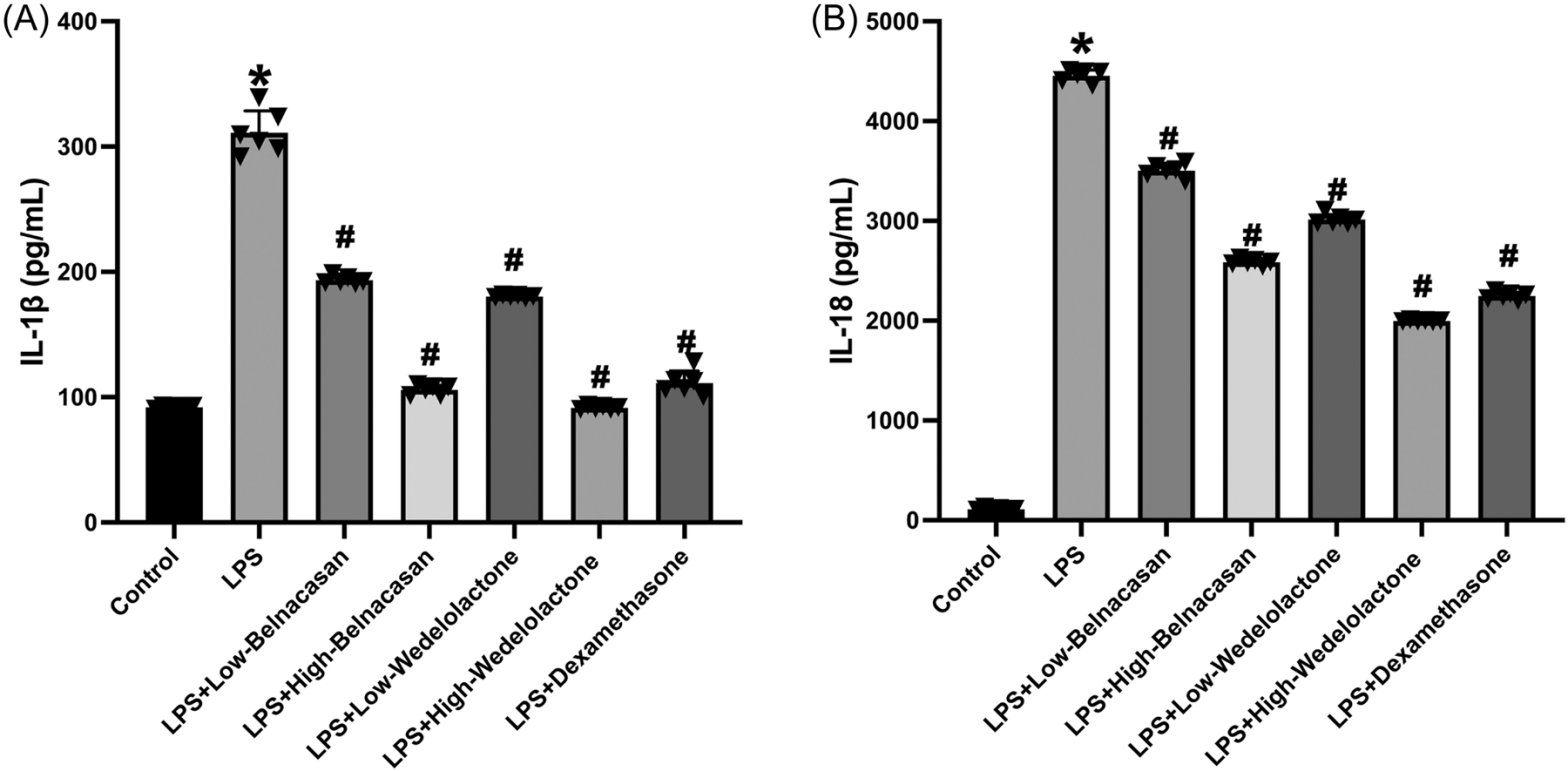
ELISA examined (A) IL‐1β and (B) IL‐18 activities in the mouse serum, *N* = 5. *p < .005 versus control, ^#^ *p* < .005 versus lipopolysaccharide.

Serum levels of IL‐1β and IL‐18 were measured to determine the impact of pyroptosis on inflammation. It can be concluded that these compounds effectively reduced the inflammatory effect of pyroptosis. Analysis using ELISA revealed that IL‐1β and IL‐18 were significantly increased in the LPS group compared to the control group. In contrast, the LPS + low Belnacasan, LPS + high Belnacasan, LPS + low Wedelolactone, LPS + high Wedelolactone, and LPS + dexamethasone groups showed decreased expressions of IL‐1β and IL‐18 relative to the LPS group (Figure 3A,B).

### 3.4 In mice with acute lung injury caused by sepsis, both classical and nonclassical pathways of pyroptosis occurred

**FIGURE 4:**
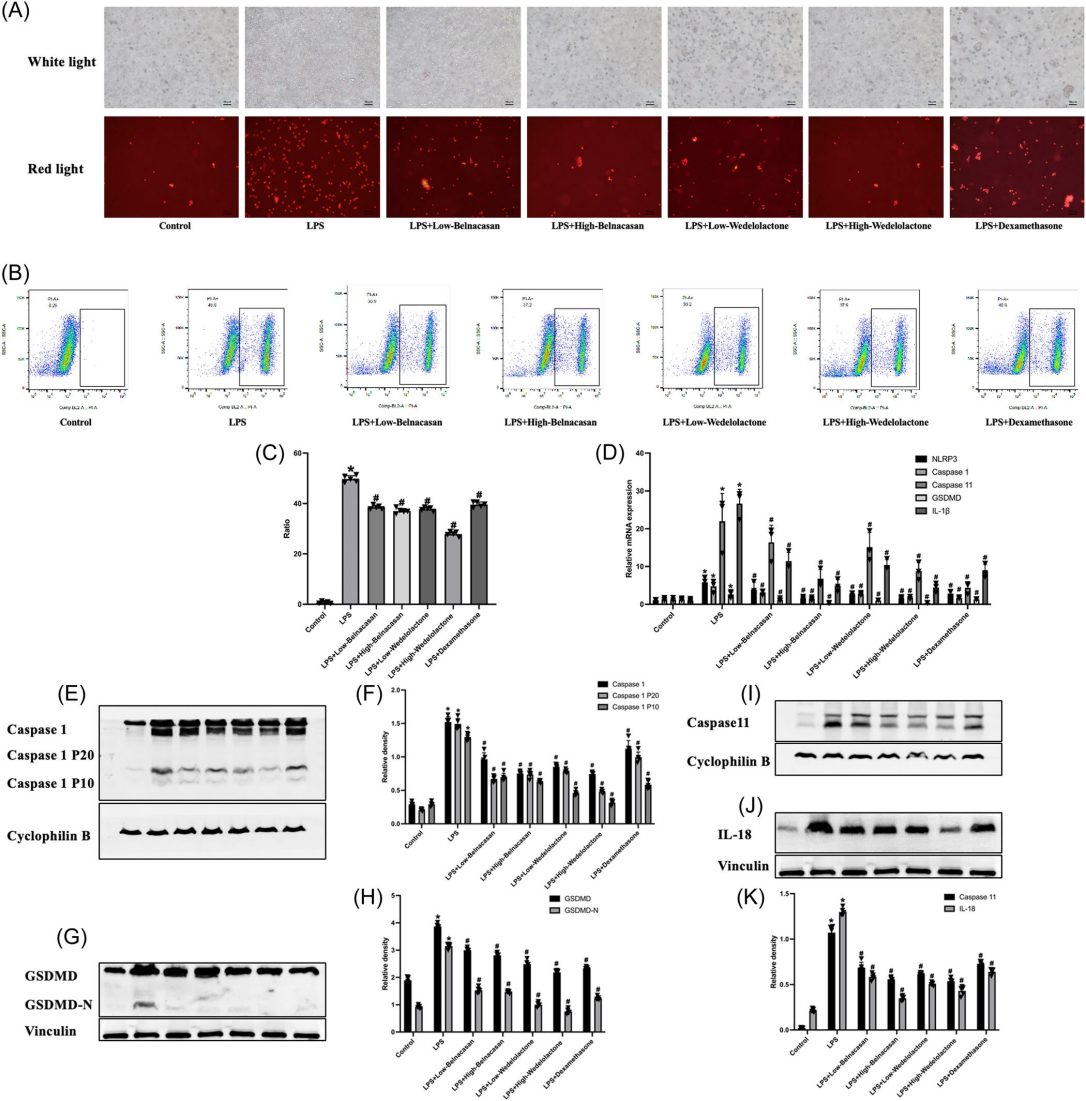
In mice with acute lung injury caused by sepsis, both classical and nonclassical pathways of pyroptosis occurred. (A) The cell graph after propidium iodide staining was observed by microscope (×200), *N* = 3. (B, C) Flow cytometric profiles of pyroptosis cells, *N* = 5. (D) Analysis of messenger RNA (mRNA) levels of NLRP3, Caspase 1, Caspase 11, GSDMD, and IL‐1β in lung. (E, F) The protein levels of Caspase 1, Caspase 1 P20, and Caspase 1 P10 in lung were measured via western blot. (G, H) The protein levels of GSDMD and GSDMD‐N in lung were measured via western blot. (I–K) The protein levels of Caspase 11 and IL‐18 in lung were measured via western blot. *p < .05 versus control, ^#^ *p* < .05 versus lipopolysaccharide.

PI staining results indicate that, compared to the control group, PI staining was strongly positive under the microscope in the LPS group. Additionally, PI staining in the LPS + low Belnacasan, LPS + high Belnacasan, LPS + low Wedelolactone, LPS + high Wedelolactone, and LPS + dexamethasone groups significantly decreased when compared to the LPS group (Figure 4A). Flow cytometry analysis revealed a significantly higher rate of alveolar macrophage pyroptosis in the LPS group when compared to the control group. However, the positive rate of alveolar macrophage pyroptosis was significantly reduced in the LPS + low Belnacasan, LPS + high Belnacasan, LPS + low Wedelolactone, LPS + high Wedelolactone, and LPS + dexamethasone groups (Figure 4B,C). The findings suggest that alveolar macrophages in mice experiencing ALI from sepsis undergo pyroptosis via the classical and nonclassical pathways. Western blot analysis revealed the presence of Caspase 1, Caspase 11, GSDMD, and IL‐18 proteins in the lung, which were consistent with the results of PI staining and flow cytometry analysis (Figure 4E–K). In addition, PCR was employed to detect mRNA changes in the lung, and the outcomes were in agreement with Western blot analysis (Figure 4D).

### 3.5 | Inhibition of pyroptosis can affect the polarization of alveolar macrophages

**FIGURE 5:**
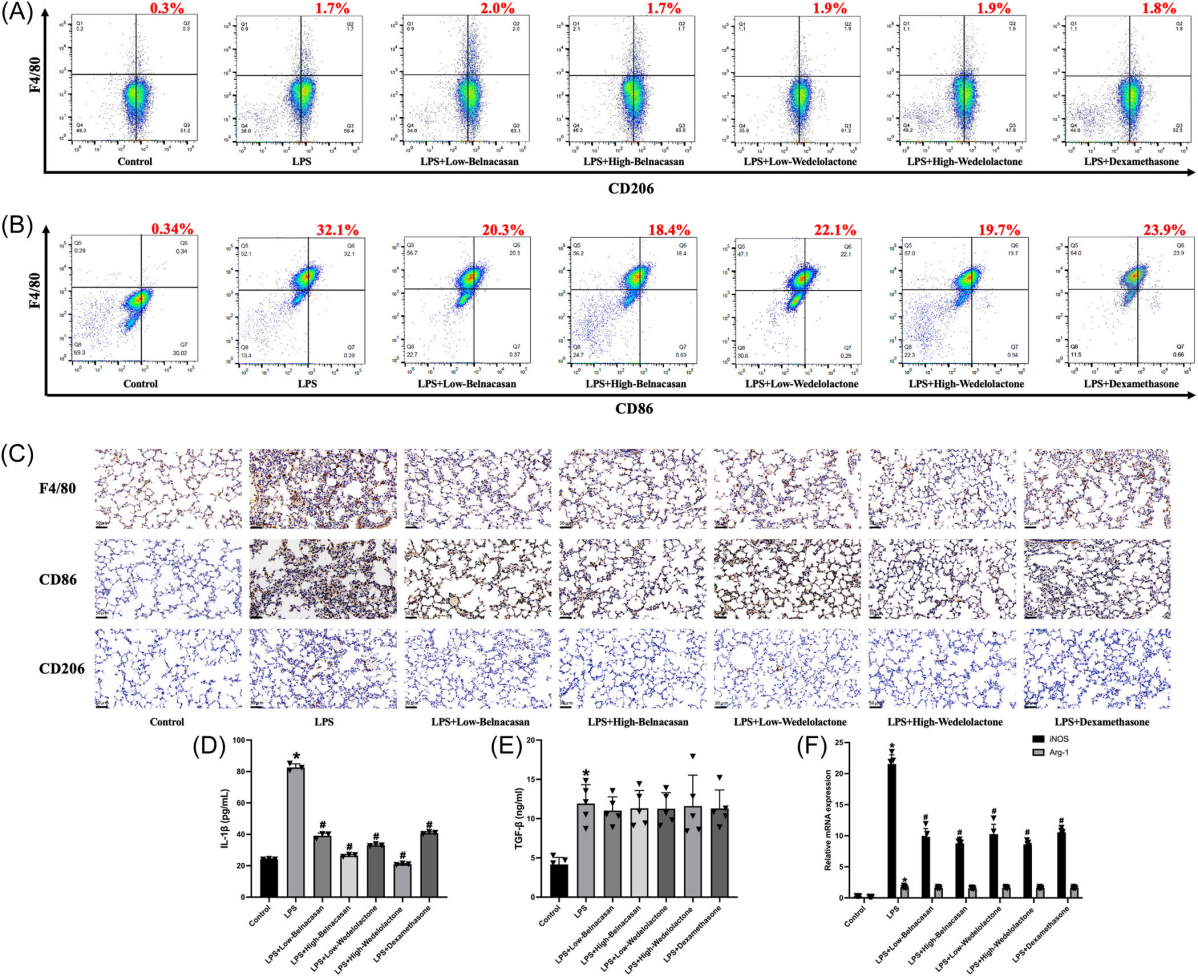
Inhibition of pyroptosis can affect the polarization of alveolar macrophages. (A) Representative images and quantitative analysis of M2 rate were assessed using flow cytometry. (B) Representative images and quantitative analysis of M1 rate were assessed using flow cytometry. (C) The immunohistochemical results of F4/80, CD86, and CD206 in the lungs (×200), *N* = 3. (D) The concentrations of IL‐1β in alveolar lavage fluid were examined with ELISA kits, *N* = 3. (E) The concentrations of TGF‐β in alveolar lavage fluid were examined with ELISA kits, *N* = 5. (F) Analysis of messenger RNA (mRNA) levels of iNOS and Arg‐1 in lung, *N* = 5. *p < .05 versus control, ^#^ *p* < .05 versus lipopolysaccharide.

To investigate the impact of pyroptosis on the polarization of alveolar macrophages, we conducted flow cytometry and immunohistochemistry. The experimental findings indicated a significant increase in both M1 and M2 macrophages in the LPS group when compared to the control group. M1 macrophages were significantly reduced in the LPS + low Belnacasan, LPS + high Belnacasan, LPS + low Wedelolactone, LPS + high Wedelolactone, and LPS + dexamethasone groups compared to the LPS group. However, there were no significant changes observed in M2 macrophages (Figure 5A–C). The results were further validated through ELISA analysis of alveolar lavage fluid and PCR analysis (Figure 5D–F).

## 4 DISCUSSION

Currently, clinical studies have advanced in understanding the pathogenesis of ALI in sepsis, including the mechanisms of inflammatory cells, coagulation systems, oxidative stress, pulmonary surfactant, and genetics. However, these studies lack detailed and in‐depth analysis of the pathogenesis.^10^, ^11^, ^12^, ^13^ Starting from the inflammatory cell mechanism, this study investigates the impact of alveolar macrophage pyroptosis on polarization during ALI caused by sepsis in mice and its corresponding mechanism. The experiment employed a mouse model of septic ALI and focused on alveolar macrophages, distinguishing between different groups. The study examined the mechanism of pyroptosis and alveolar macrophage polarization in mice with sepsis‐induced ALI using diverse experimental approaches at varying levels. Previous in vitro research affirmed the occurrence of pyroptosis in M0, M1, and M2 macrophages, with M1 displaying the most significant effect, trailed by M2. Based on our previous research, in this experiment, we used a mouse model of ALI with sepsis as a carrier. The NLRP3/Caspase/GSDMD signaling pathway of sepsis‐induced ALI in mice caused by pyroptosis was assessed by real‐time quantitative PCR, enzyme‐linked immunoadsorption assay, western blot analysis, PI staining, and flow cytometry. Expression levels of the signature molecule CD86 on the surface of M1 macrophages and CD206 on the surface of M2 macrophages were determined by immunohistochemical staining and flow cytometry. In addition, the relative expression of iNOS and Arg‐1 mRNA by quantitative PCR confirmed the polarization of alveolar macrophages in mice with ALI caused by sepsis, and we found that intervention with pyroptosis inhibitor could alter the polarization of alveolar macrophages and reduce the number of M1‐type alveolar macrophages. The results of this study suggest that both classical and nonclassical pyroptosis can occur in ALI in septic mice and that pyroptosis inhibitors can ameliorate ALI in septic mice. In mice with ALI caused by sepsis, the polarization of alveolar macrophages occurred simultaneously as pyroptosis, and pyroptosis inhibitors could affect the direction of polarization. Pyroptosis of alveolar macrophages in ALI of septic mice can cause polarization of alveolar macrophages toward the M1 type, promoting lung inflammation and destroying the immune balance environment of the body. This is consistent with our previous findings that M0, M1, and M2 macrophages can all induce pyroptosis, with M1 being the most significant, followed by M2, confirming our hypothesis based on previous in vitro studies.

Unlike previous studies, this is the first time that we have investigated the interaction mechanism between pyroptosis and alveolar macrophage polarization in a mouse model of ALI with sepsis, which is the innovation of this study. By using the mouse model of ALI with sepsis as a carrier, the relationship between pyroptosis and polarization is closer to the internal environment during the onset and development of the disease than the previous in vitro cell experiments, and the mechanism of the effect of pyroptosis on polarization in the development of lung inflammation in ALI with sepsis is better explored. As a severe systemic inflammatory response syndrome, sepsis‐induced ALI is a major cause of death in patients with sepsis. Although there are many therapeutic options for patients with ALI due to sepsis, the therapeutic effect is still not good.^13^ The completion of this project will, to a certain extent, reveal the mechanism of pyroptosis of different alveolar macrophages in ALI in sepsis, provide a certain theoretical basis for the occurrence and development mechanism of ALI in sepsis, and lay a certain preliminary foundation for further clinical exploration of the pathogenesis, regulatory mechanism, and precise targeted treatment of ALI in sepsis.

There are several limitations to the study. First, ALI in sepsis is a dynamic developmental process that constantly changes, evolves and transforms under different environmental stimuli. This study only examined the point in time after the 8‐h intervention with LPS and could not monitor the dynamic development of the disease or observe the changes and interactions of pyroptosis and polarization in a shorter or longer period of time. In future research, we can further explore the mechanism based on the time course. Secondly, there is a certain gap between animal studies and clinical studies, and the conclusions drawn in animal studies may differ from the results of clinical studies. We need to include data from patients with ALI in clinical sepsis in future studies to make our conclusions more reliable.

Pyroptosis and polarization of alveolar macrophages are closely related to the occurrence and development of the pulmonary inflammatory response. In an uncontrolled state, inflammatory factor storms can occur in the body, resulting in a cascade amplification of the inflammatory response and exacerbating the degree of lung injury.^15^, ^16^ Pyroptosis and polarization have a dual effect on the body. At different stages, they either enhance the body's defences and play a protective role, or they destroy the body's immune function and cause damage to the body. To date, studies have shown that pyroptosis plays an important role in the onset and development of inflammatory diseases, cardiovascular diseases, sepsis, cancer, immune diseases, metabolic diseases, and other diseases. At the same time, the signaling pathway of pyroptosis in the occurrence and development of diseases has been clarified, and drugs that can inhibit pyroptosis to achieve the therapeutic effect of diseases have been found.^2^, ^17^, ^18^, ^19^, ^20^, ^21^ At present, the pathogenesis and regulatory mechanism of pyroptosis are relatively clear, and there are complex regulatory networks and mechanisms of alveolar macrophage polarization. Based on the results of this study, we believe that the direction of alveolar macrophage polarization can be changed by regulating alveolar macrophage pyroptosis. As an important player in maintaining lung tissue homeostasis and regulating the immune response, the ability of alveolar macrophages to change their polarization state in time in response to environmental stimuli is important for their heterogeneity and function. Future studies can regulate the pyroptosis pathway of M1 alveolar macrophages through targeted inhibitors, restore immune balance, and reduce tissue inflammation and injury during sepsis‐associated ALI, which will further elucidate the occurrence and development mechanism of sepsis‐associated ALI and highlight its potential as a treatment strategy for sepsis‐associated ALI. It provides an effective theoretical basis for accurate and targeted treatment of ALI in sepsis.

## 5 CONCLUSION

In summary, it was discovered that pyroptosis of alveolar macrophages in mice with sepsis‐induced ALI can trigger polarization of alveolar macrophages to the M1 type. Our study provides a reliable theoretical foundation for the treatment of sepsis‐induced ALI.

## AUTHOR CONTRIBUTIONS

Sijiang Zhou, Xia Yang, Kanglin Mo and Zong Ning performed the research. Zong Ning and Kanglin Mo designed the research study. Xia Yang contributed essential reagents or tools. Sijiang Zhou analyzed the data. Sijiang Zhou, Xia Yang, Kanglin Mo, and Zong Ning wrote the paper. All authors have read and approved the final manuscript.

## CONFLICT OF INTEREST STATEMENT

The authors declare no conflict of interest.

## ETHICS STATEMENT

All animal procedures were operated in light of the NIH Guide for the Care and Use of Laboratory Animals, approved by the ethical guidelines of Guangxi Medical University, and were conducted in light of the ARRIVE guidelines. All the authors agreed to be published.

## Data Availability

The analyzed data sets generated during the present study are available from the corresponding author on reasonable request.
